# Supplementary material for: Glycolipids Derived from the Korean Endemic Plant Aruncus aethusifolius Inducing Glucose Uptake in Mouse Skeletal Muscle C2C12 Cells
Source: Plants (Basel). 2024 Feb 23;13(5):608. doi: 10.3390/plants13050608 (PMC10934126; doi:10.3390/plants13050608)
Supplement: Supplementary file 1 [file plants-13-00608-s001.zip › plants-2807398-supplementary.pdf]

plants

Article

Glycolipids Derived from the Korean Endemic Plant *Aruncus aethusifolius* Inducing Glucose Uptake in Mouse Skeletal Muscle C2C12 Cells

Jong Gwon Baek [1,2,†], Do Hwi Park [3,†], Ngoc Khanh Vu [1,†], Charuvaka Muvva [1], Hoseong Hwang [1], Sungmin Song [1], Hyeon-Seong Lee [1], Tack-Joong Kim [2], Hak Cheol Kwon [1,2], Keunwan Park [1,*], Ki Sung Kang [3,*] and Jaeyoung Kwon [1,4,*]

List of supporting information

Figure S1. ^{1}H NMR spectrum of compound **1** (500 MHz, methanol-*d*$_4$).
Figure S2. ^{13}C NMR spectrum of compound **1** (125 MHz, methanol-*d*$_4$).
Figure S3. HSQC spectrum of compound **1** (methanol-*d*$_4$).
Figure S4. HMBC spectrum of compound **1** (methanol-*d*$_4$).
Figure S5. ^{1}H-^{1}H COSY spectrum of compound **1** (methanol-*d*$_4$).
Figure S6. DEPT spectrum of compound **1** (methanol-*d*$_4$).
Figure S7. HRESIMS spectrum of compound **1**.
Figure S8. ^{1}H NMR spectrum of compound **2** (500 MHz, methanol-*d*$_4$).
Figure S9. ^{13}C NMR spectrum of compound **2** (125 MHz, methanol-*d*$_4$).
Figure S10. HSQC spectrum of compound **2** (methanol-*d*$_4$).
Figure S11. HMBC spectrum of compound **2** (methanol-*d*$_4$).
Figure S12. ^{1}H-^{1}H COSY spectrum of compound **2** (methanol-*d*$_4$).
Figure S13. HRESIMS spectrum of compound **2**.
Figure S14. ^{1}H NMR spectrum of compound **2-1** (500 MHz, methanol-*d*$_4$).
Figure S15. ^{13}C NMR spectrum of compound **2-1** (125 MHz, methanol-*d*$_4$).
Figure S16. HSQC spectrum of compound **2-1** (methanol-*d*$_4$).
Figure S17. HMBC spectrum of compound **2-1** (methanol-*d*$_4$).
Figure S18. ^{1}H-^{1}H COSY spectrum of compound **2-1** (methanol-*d*$_4$).
Figure S19. DEPT spectrum of compound **2-1** (methanol-*d*$_4$).
Figure S20. HRESIMS spectrum of compound **2-1**.
Figure S21. Sugar determination of compounds **1** and **2** by HPLC analysis.
Figure S22. Cytotoxicity test of fractions and compounds

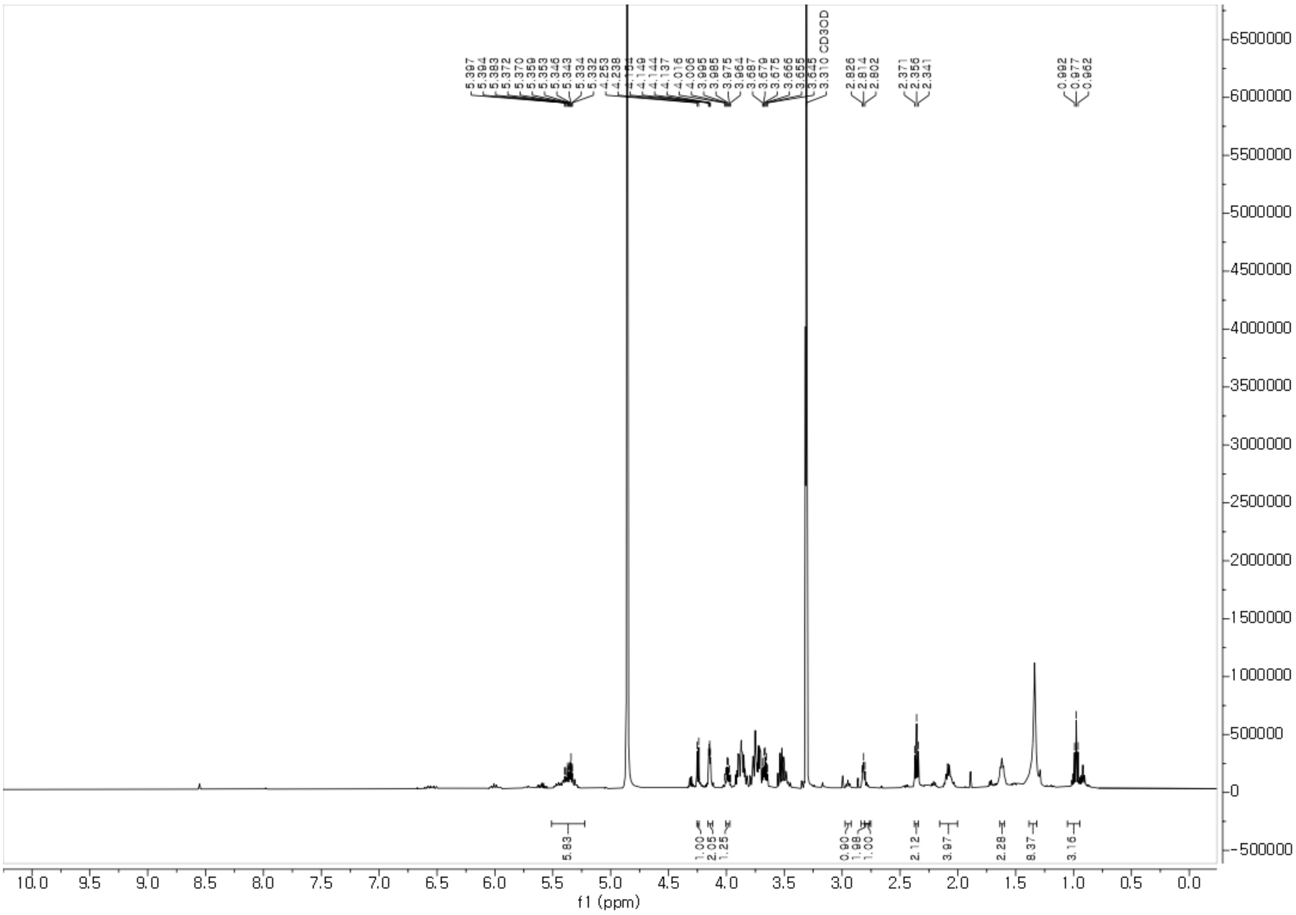

Figure S1. ^{1}H NMR spectrum of compound **1** (500 MHz, methanol-d_4).

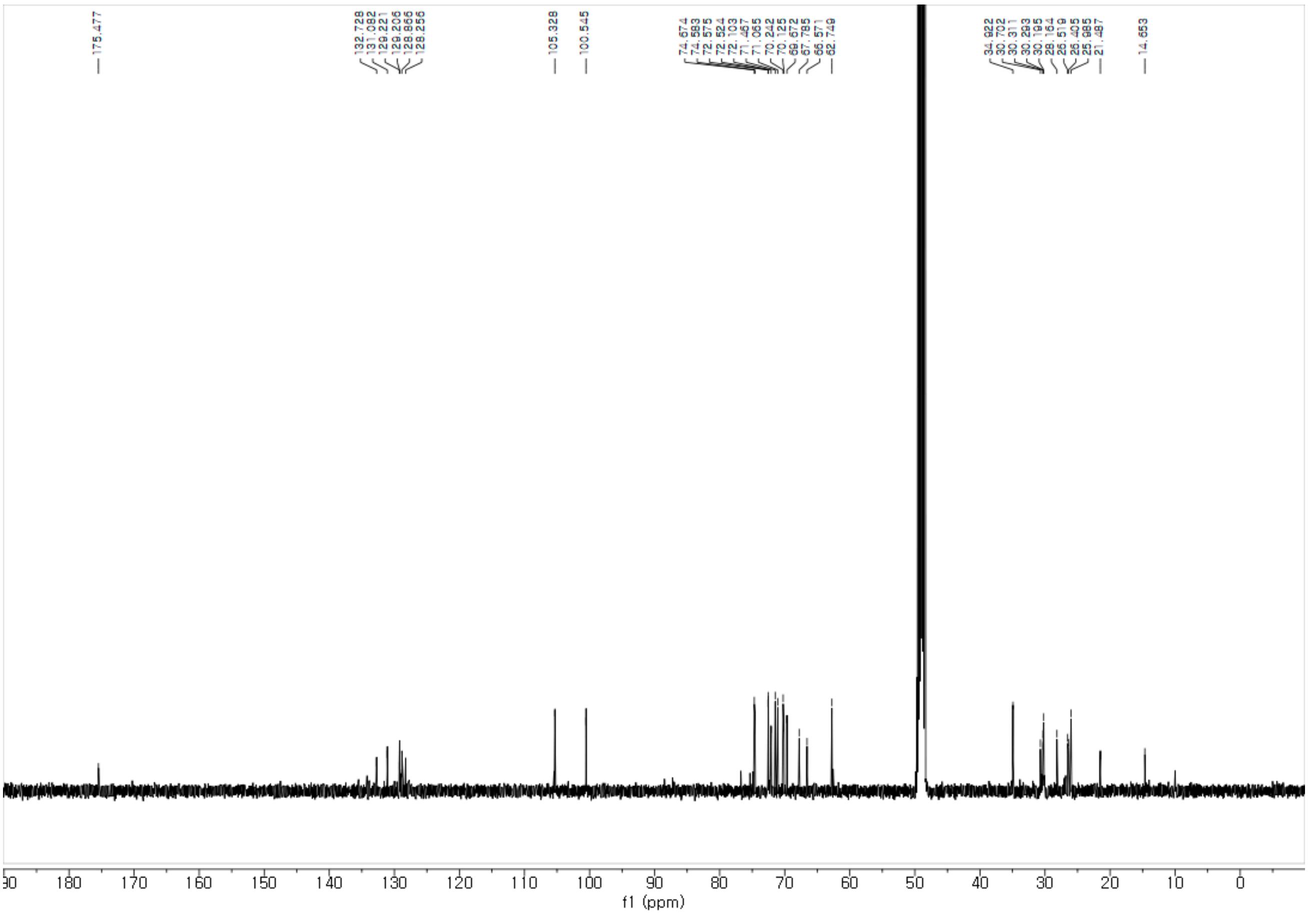

Figure S2. ^{13}C NMR spectrum of compound **1** (125 MHz, methanol-d_4).

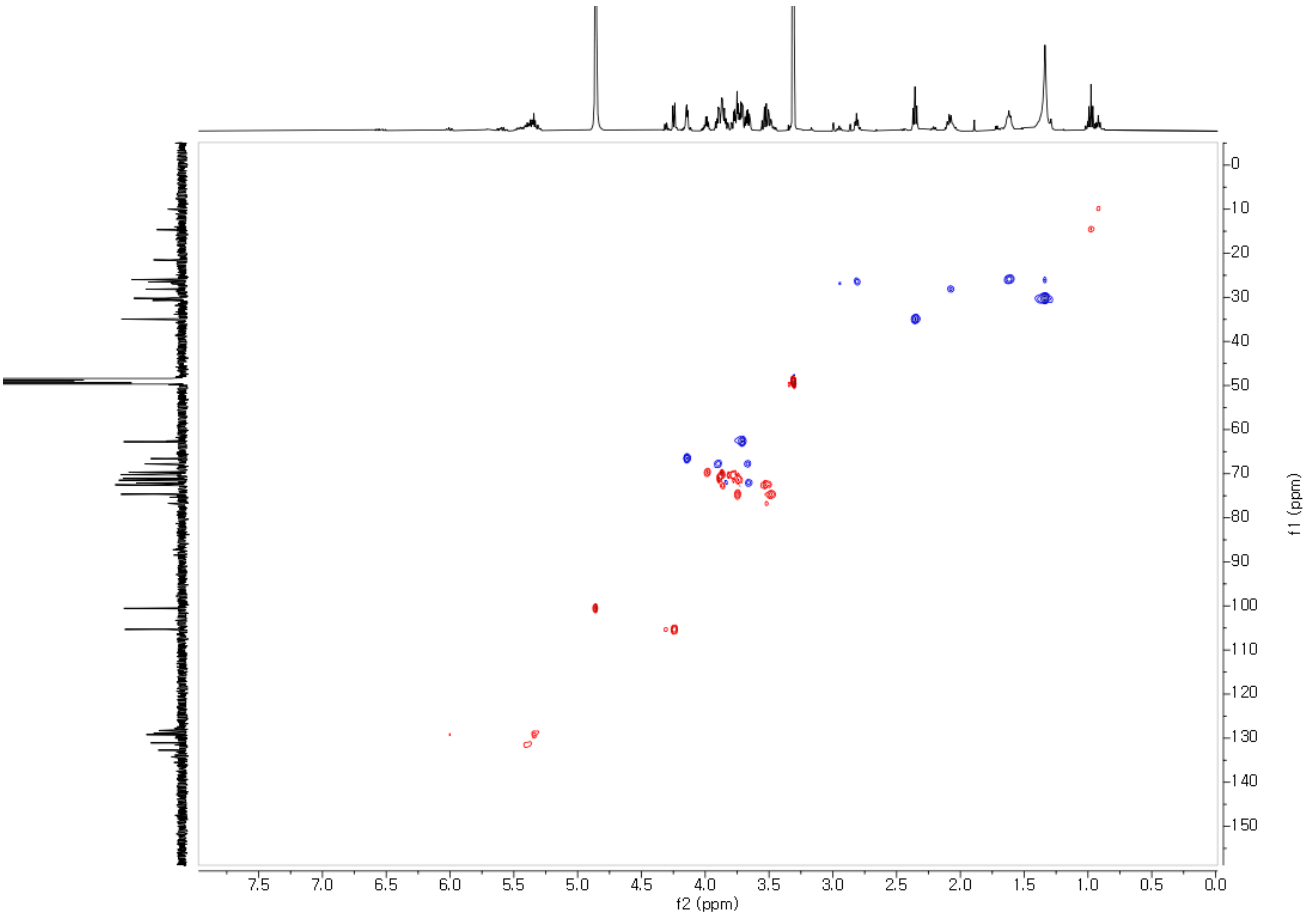

Figure S3. HSQC spectrum of compound **1** (methanol-*d*4).

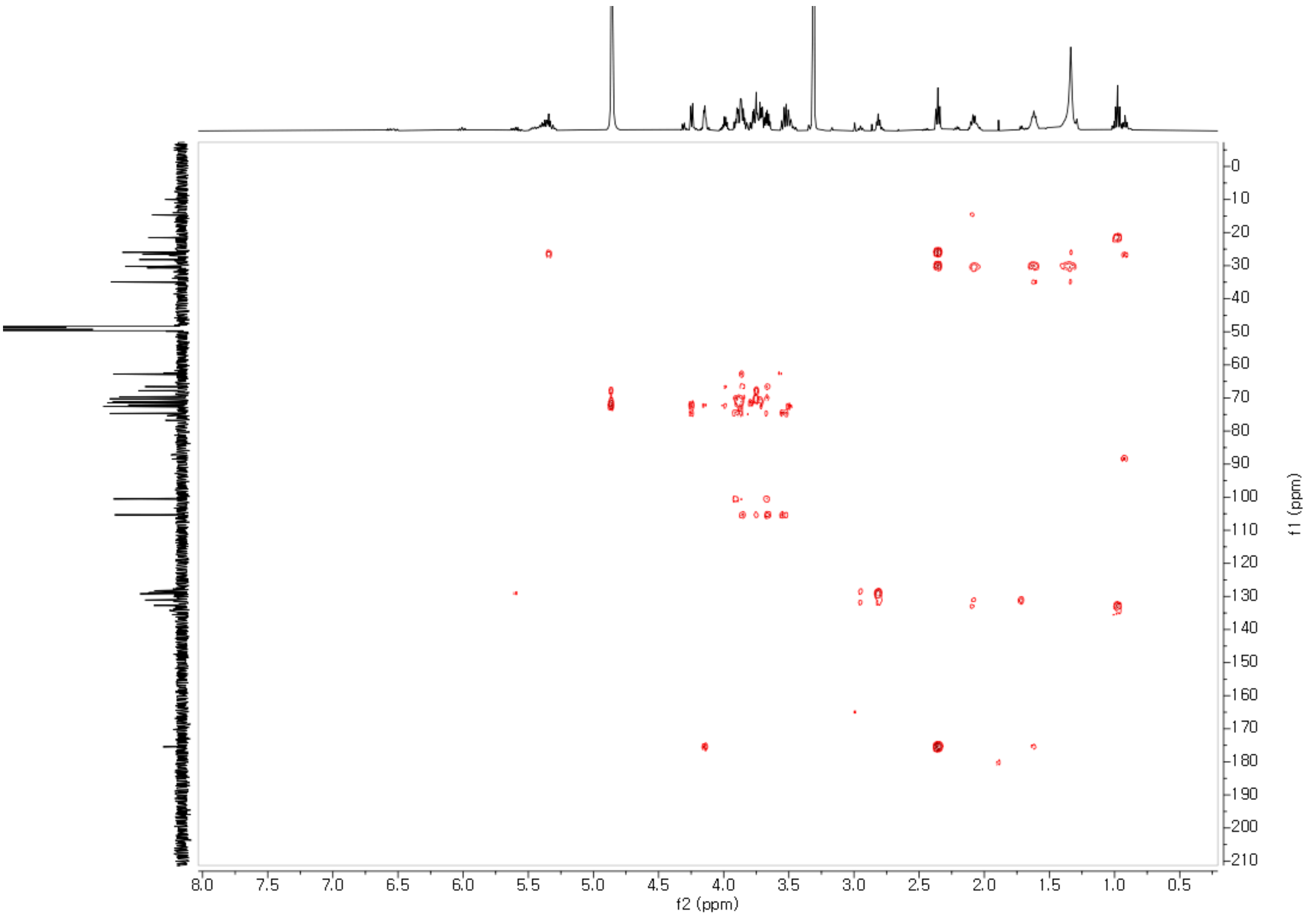

Figure S4. HMBC spectrum of compound **1** (methanol-d_4).

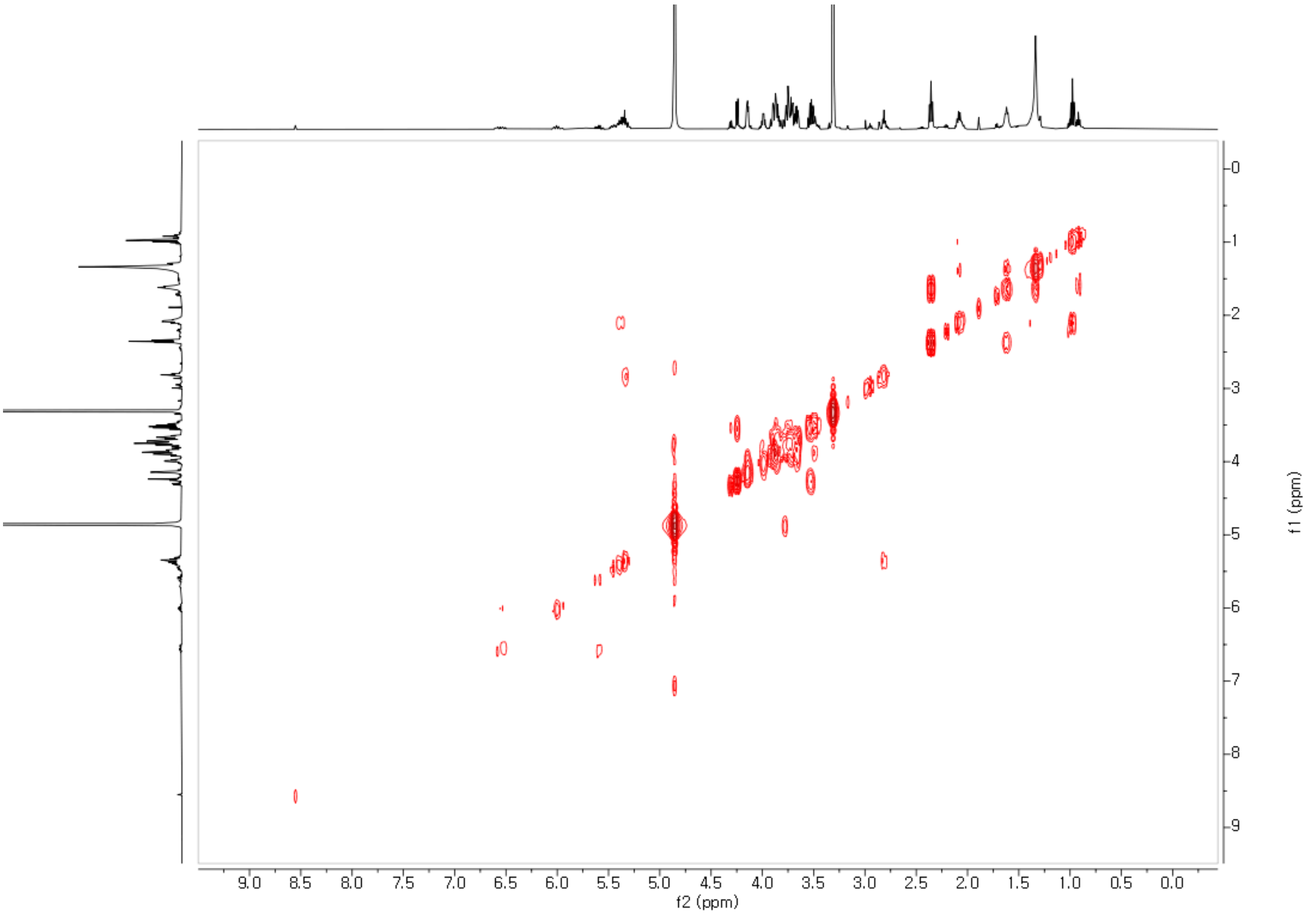

Figure S5. ^{1}H-^{1}H COSY spectrum of compound **1** (methanol-*d*$_4$).

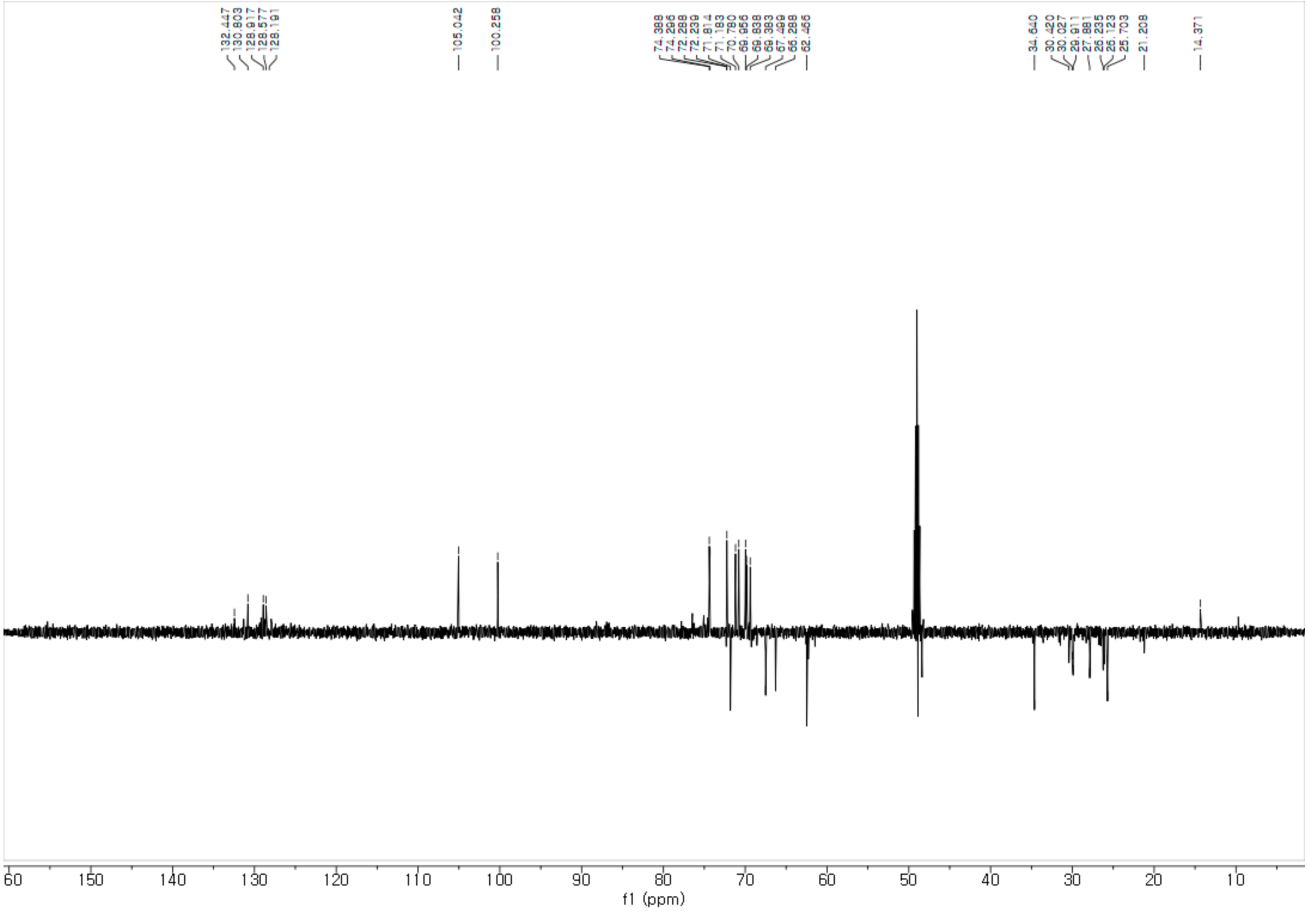

Figure S6. DEPT spectrum of compound **1** (methanol-*d*4).

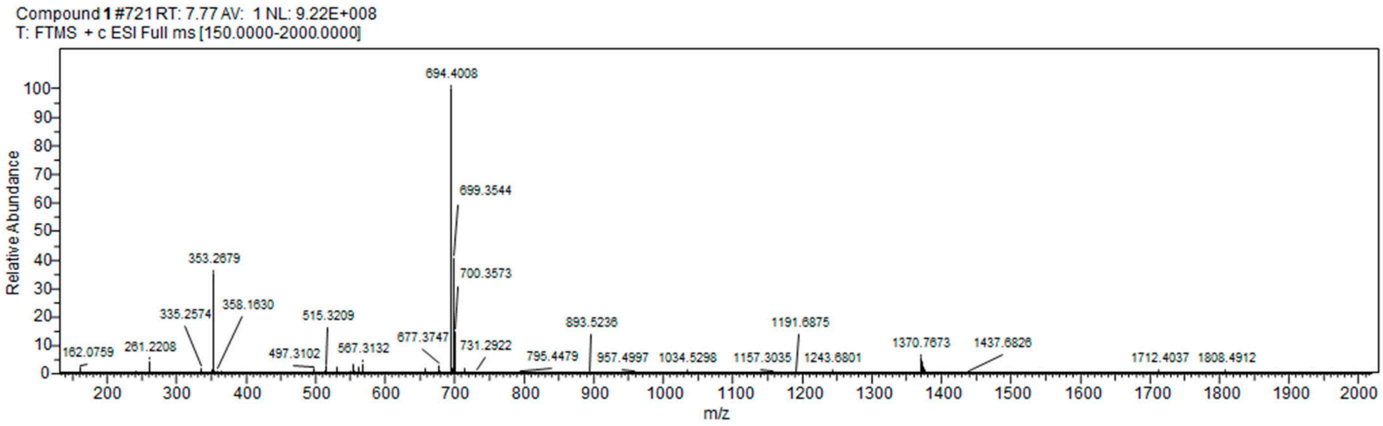

Peak Mass	Display Formula	Combined Fit	RDB	Delta [ppm]	Theo. mass	Rank	Combined Score	# Matched Iso.	# Missed Iso.	MS Cov. [%]	Pattern Cov. [%]
699.3544	$C_{33}H_{56}O_{14}{}^{23}Na$	51.68	5.50	-2.66	699.35623	1	97.37	5	0	99.91	100.00

Figure S7. HRESIMS spectrum of compound **1**.

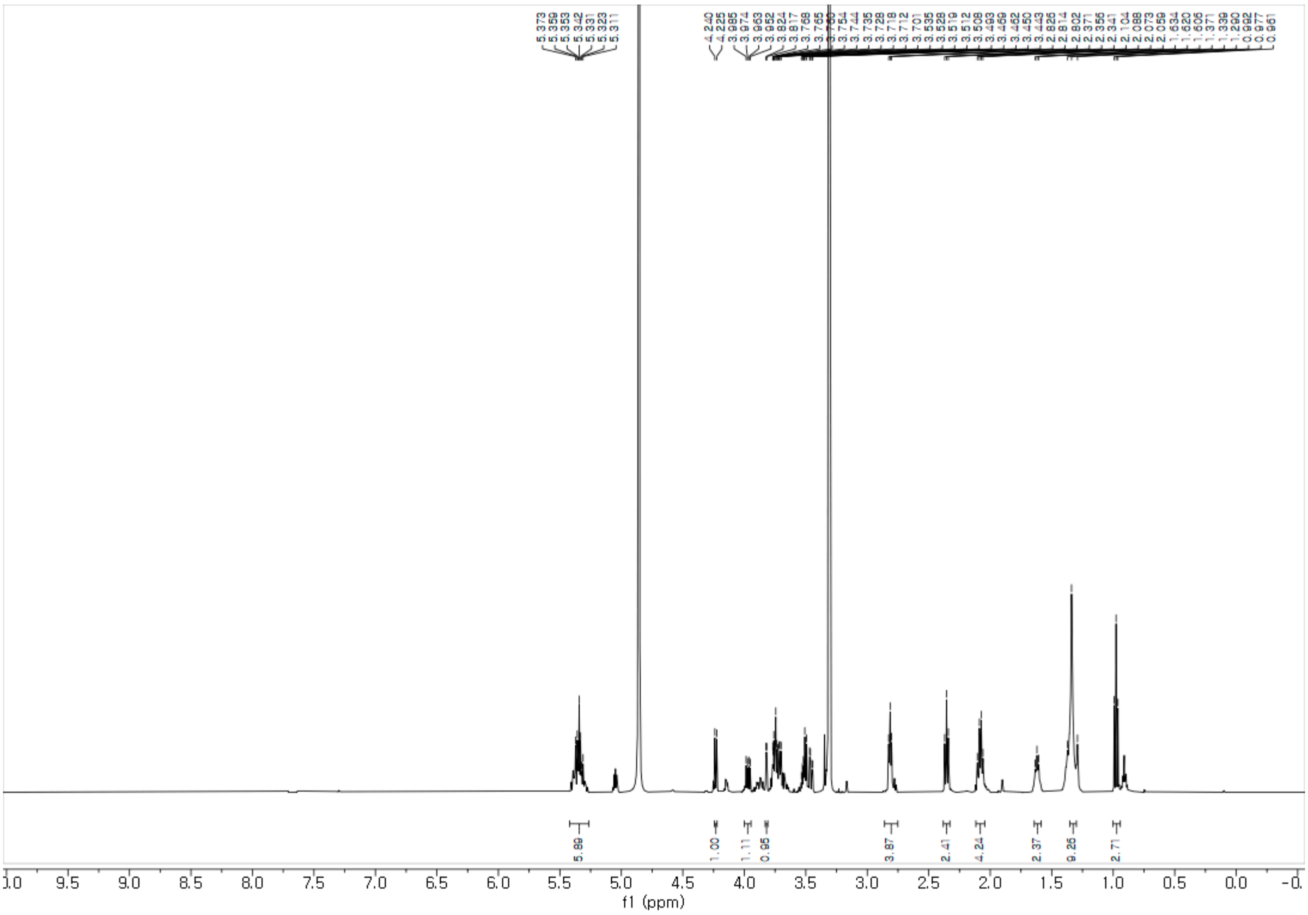

Figure S8. ^{1}H NMR spectrum of compound **2** (500 MHz, methanol-d_4).

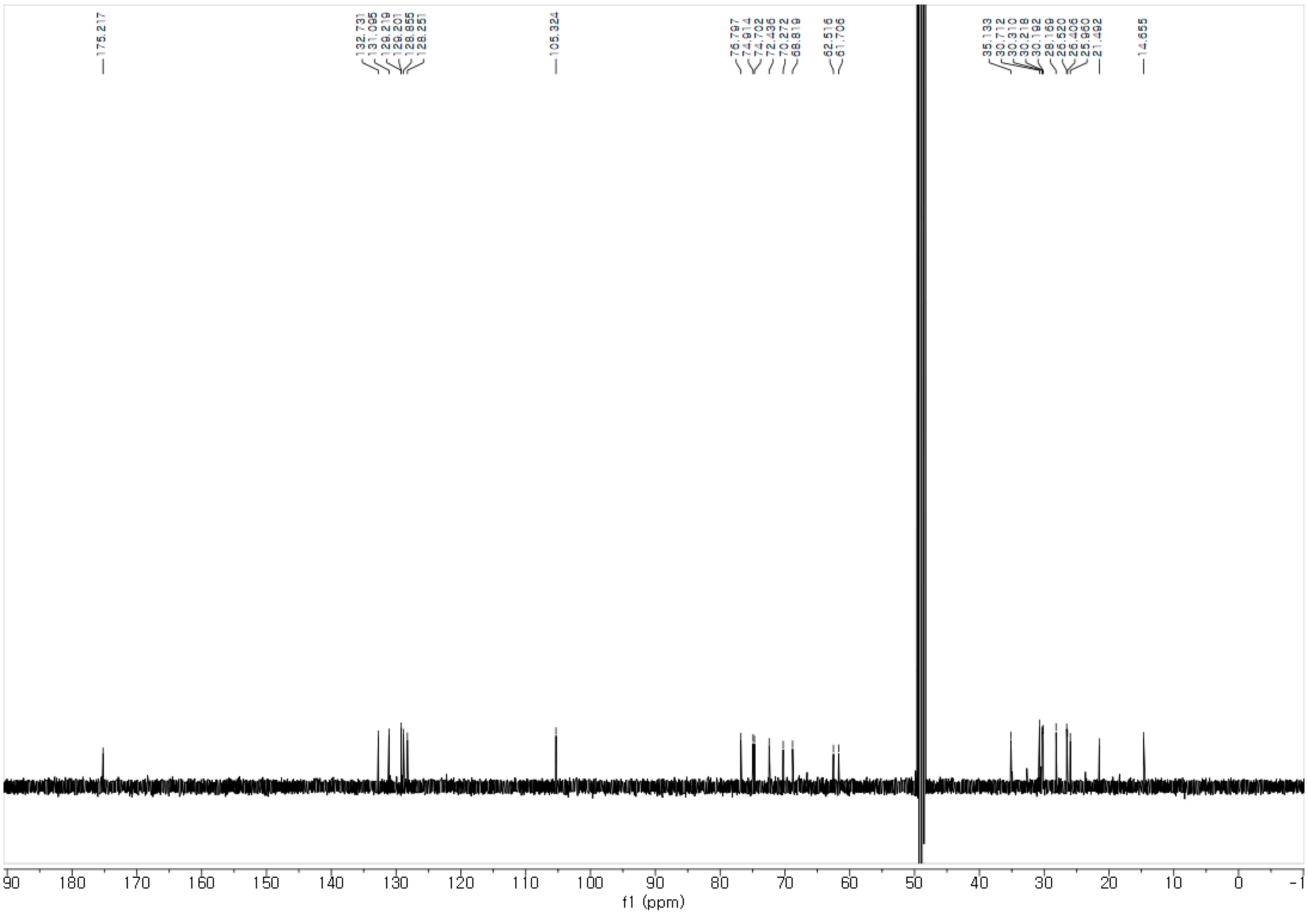

Figure S9. ^{13}C NMR spectrum of compound **2** (125 MHz, methanol-d_4).

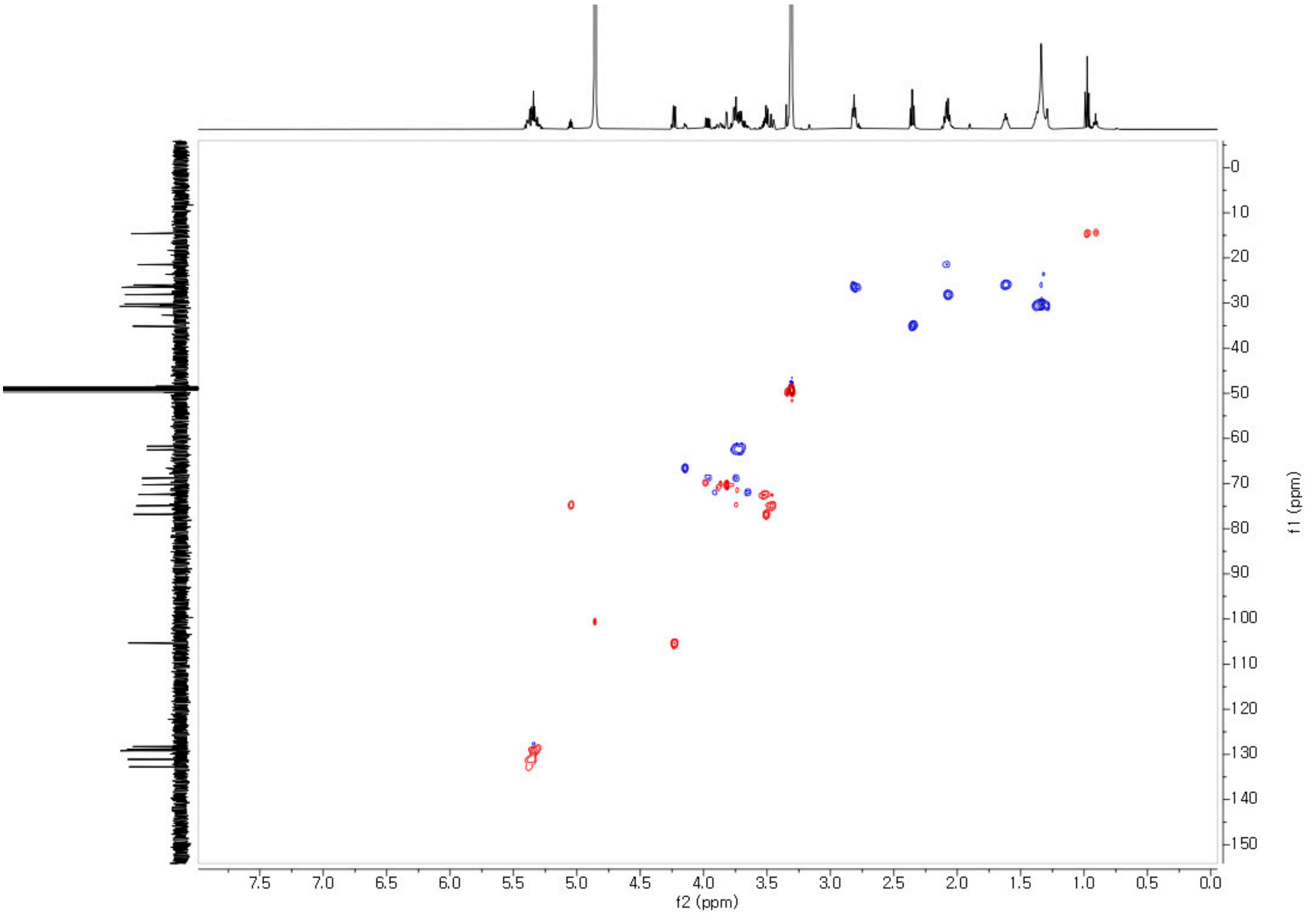

Figure S10. HSQC spectrum of compound **2** (methanol-d_4).

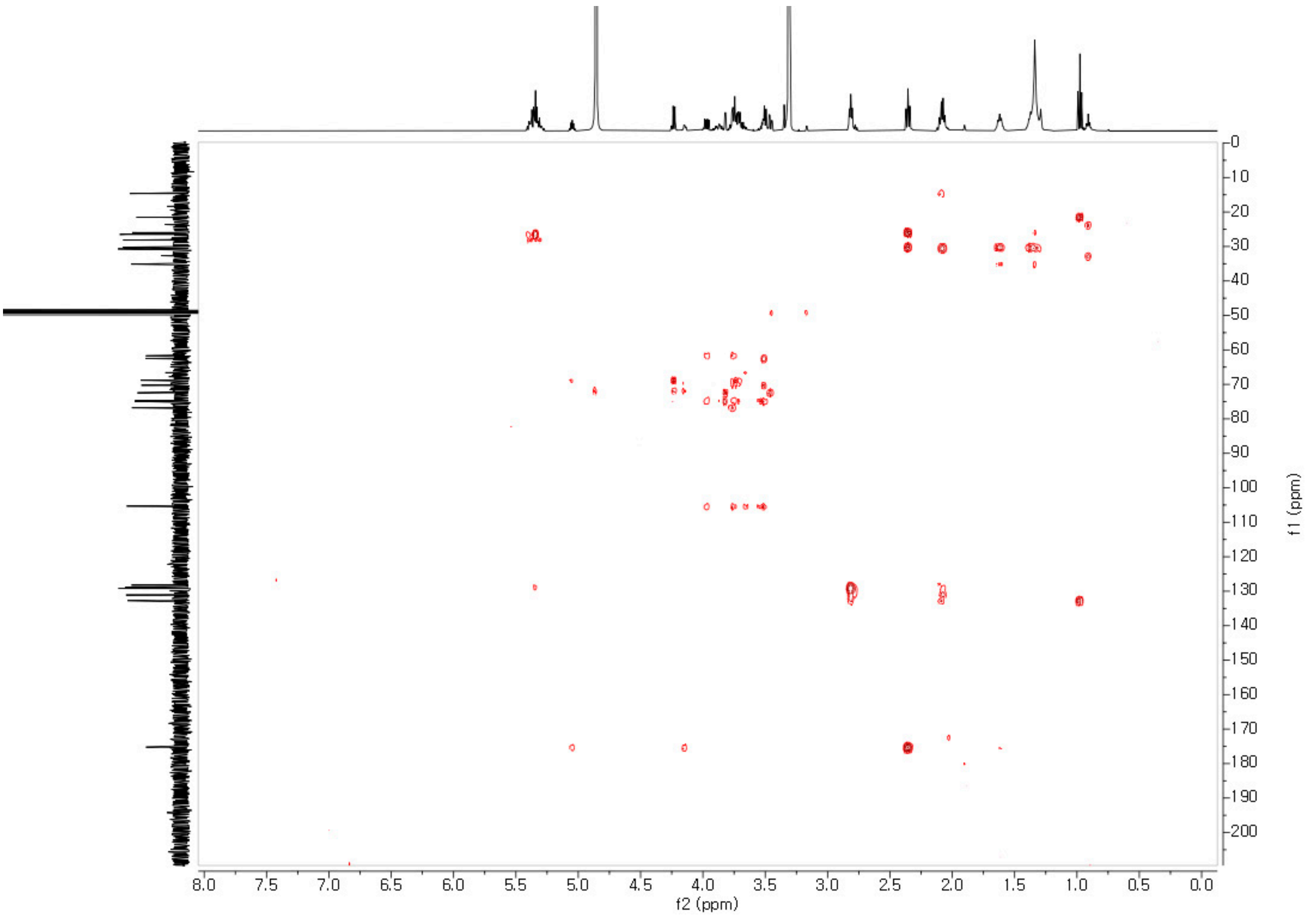

Figure S11. HMBC spectrum of compound **2** (methanol-d_4).

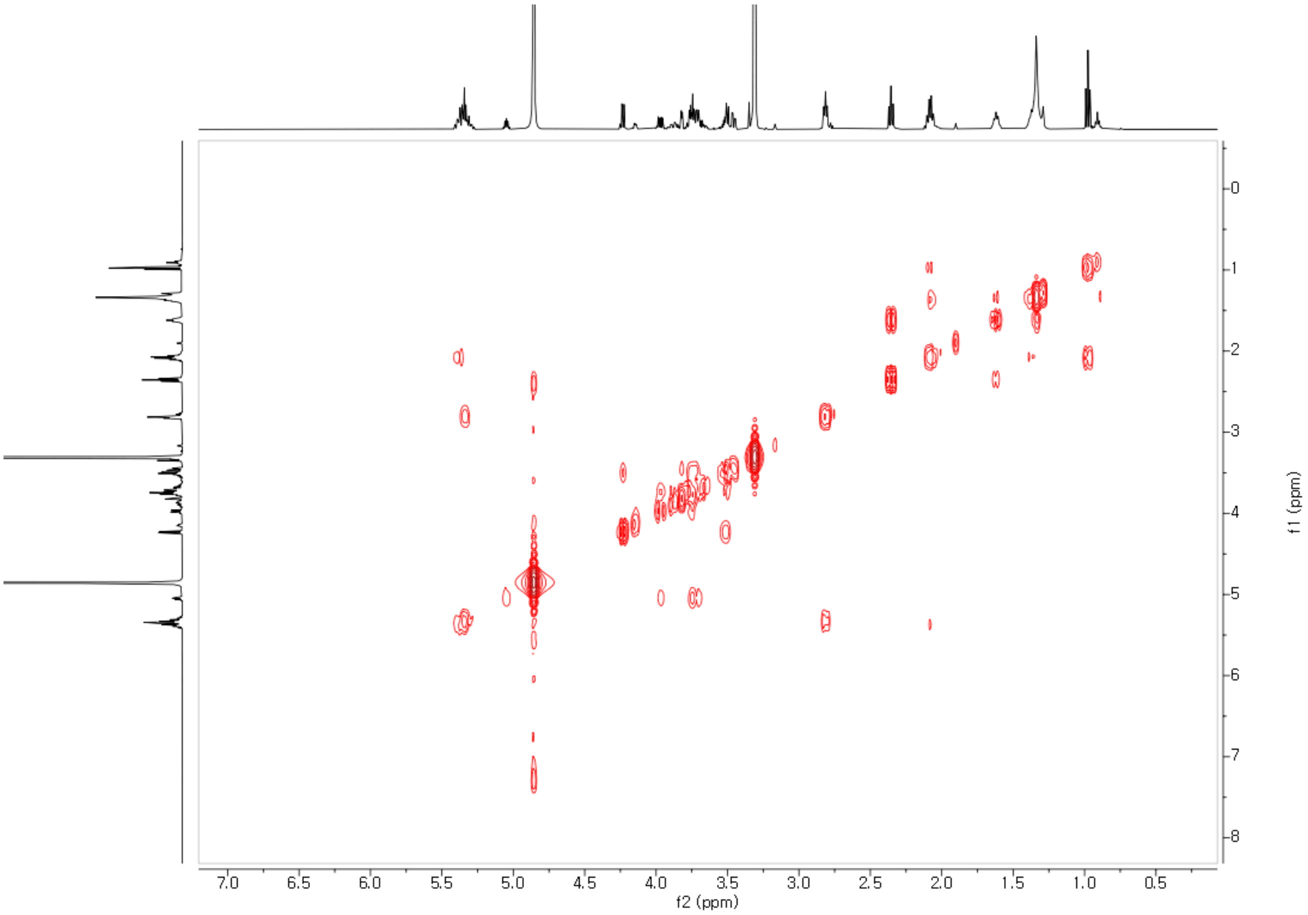

Figure S12. ^{1}H-^{1}H COSY spectrum of compound **2** (methanol-d_4).

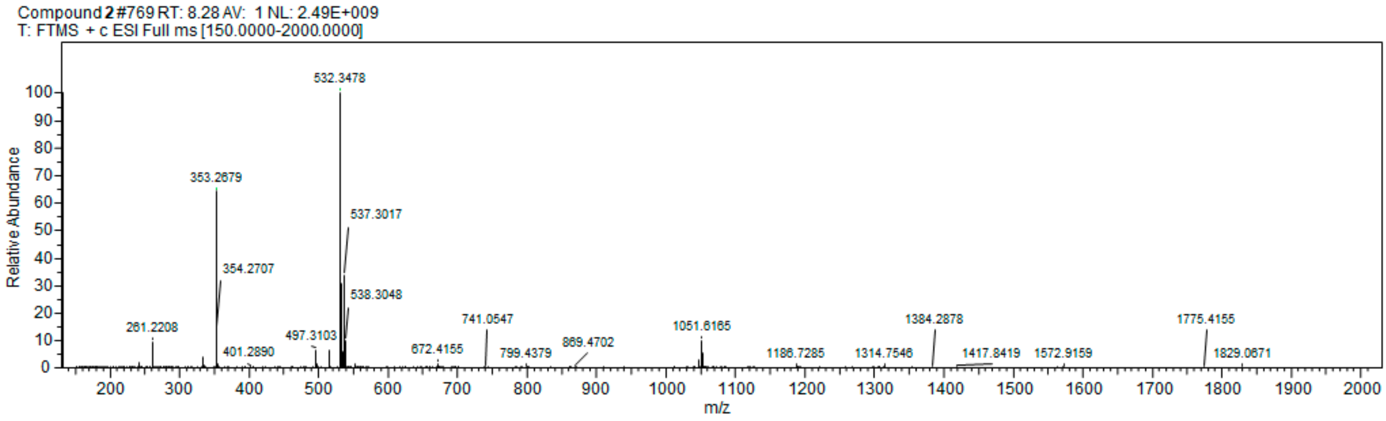

Peak Mass	Display Formula	Combined Fit	RDB	Delta [ppm]	Theo. mass	Rank	Combined Score	# Matched Iso.	# Missed Iso.	MS Cov. [%]	Pattern Cov. [%]
537.3017	$C_{27}H_{46}O_9{}^{23}Na$	47.13	4.50	-3.18	537.30340	1	97.16	5	0	99.94	100.00

Figure S13. HRESIMS spectrum of compound **2**.

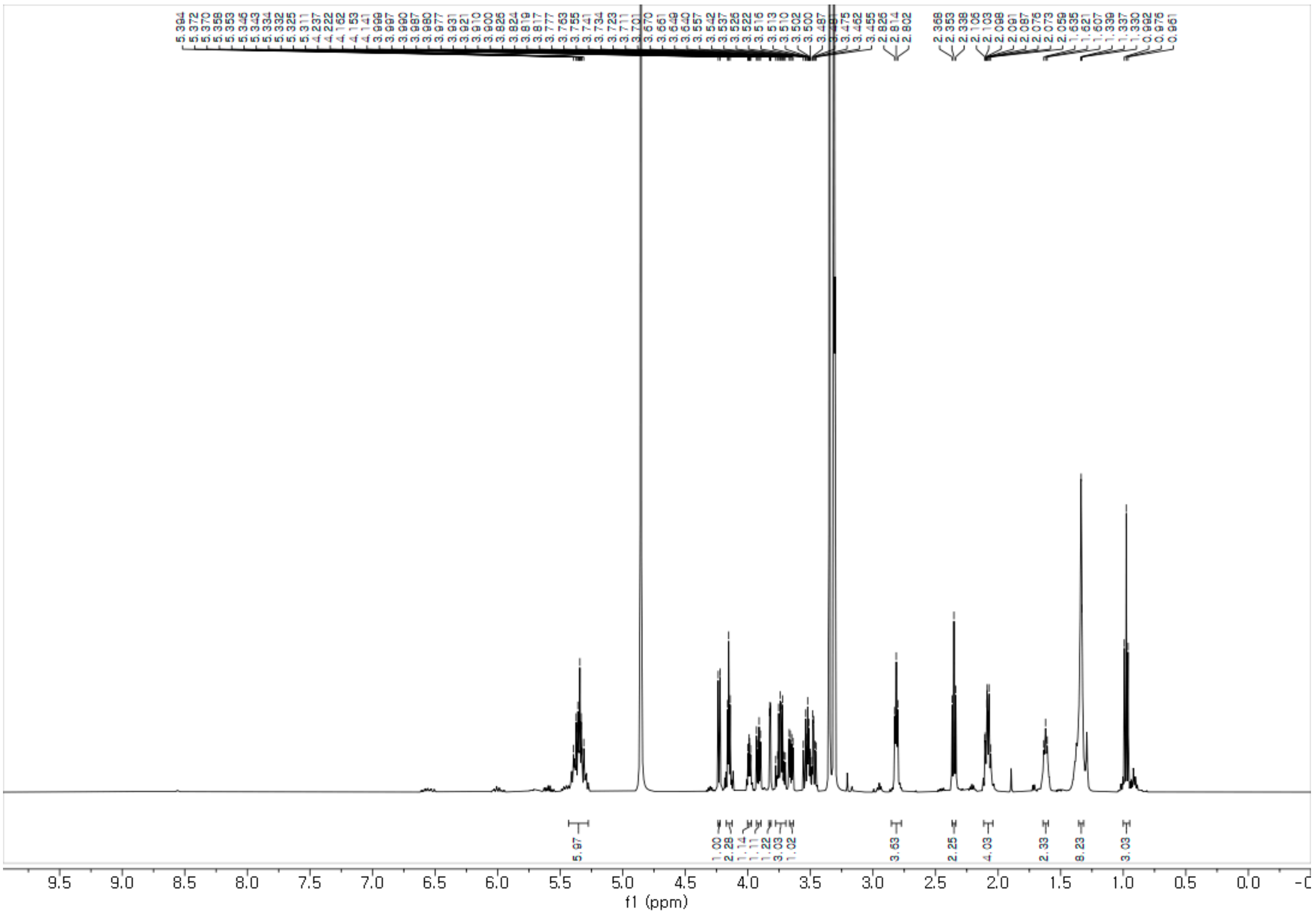

Figure S14. ^{1}H NMR spectrum of compound **2-1** (500 MHz, methanol-d_4).

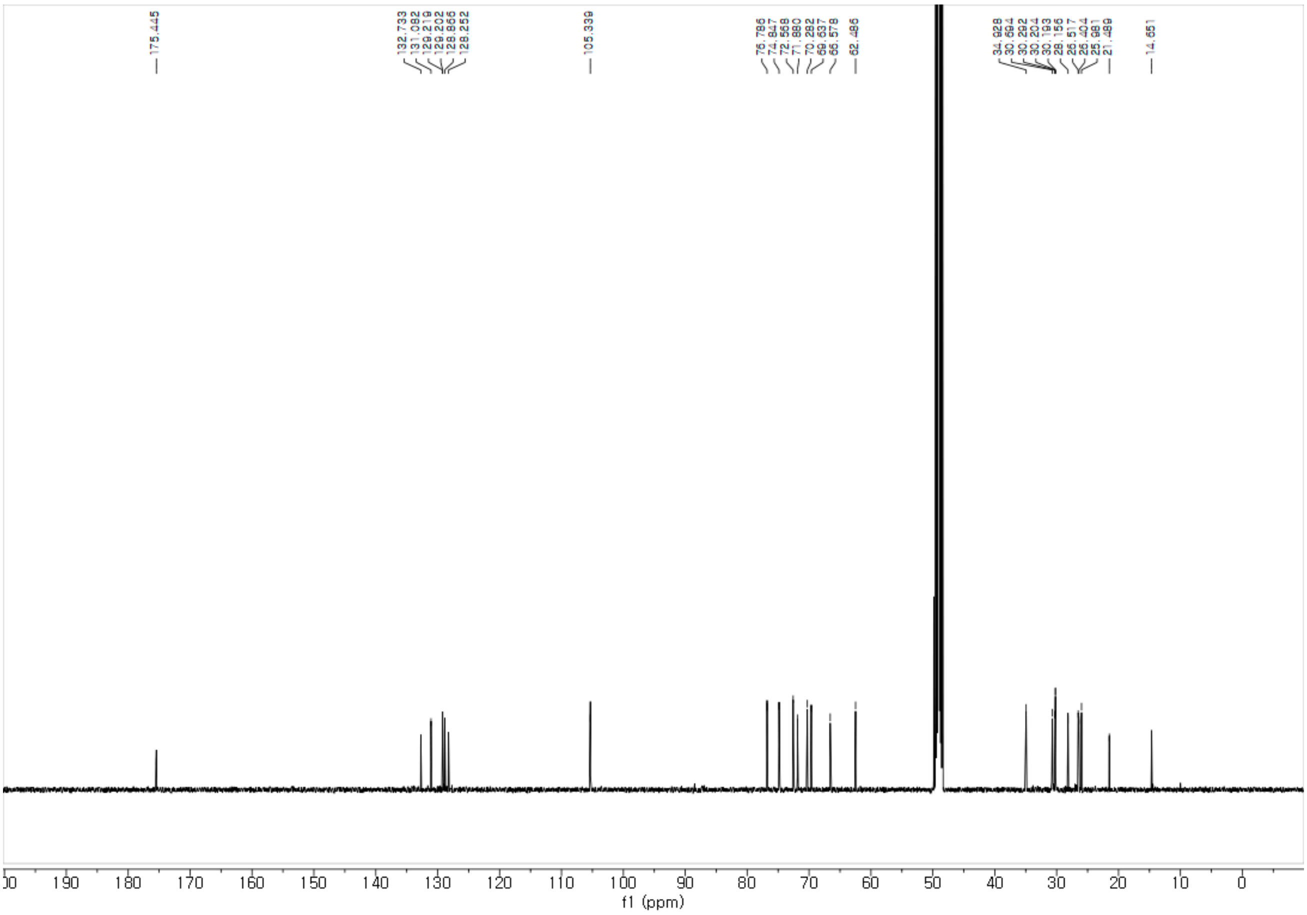

Figure S15. ^{13}C NMR spectrum of compound **2-1** (125 MHz, methanol-d_4).

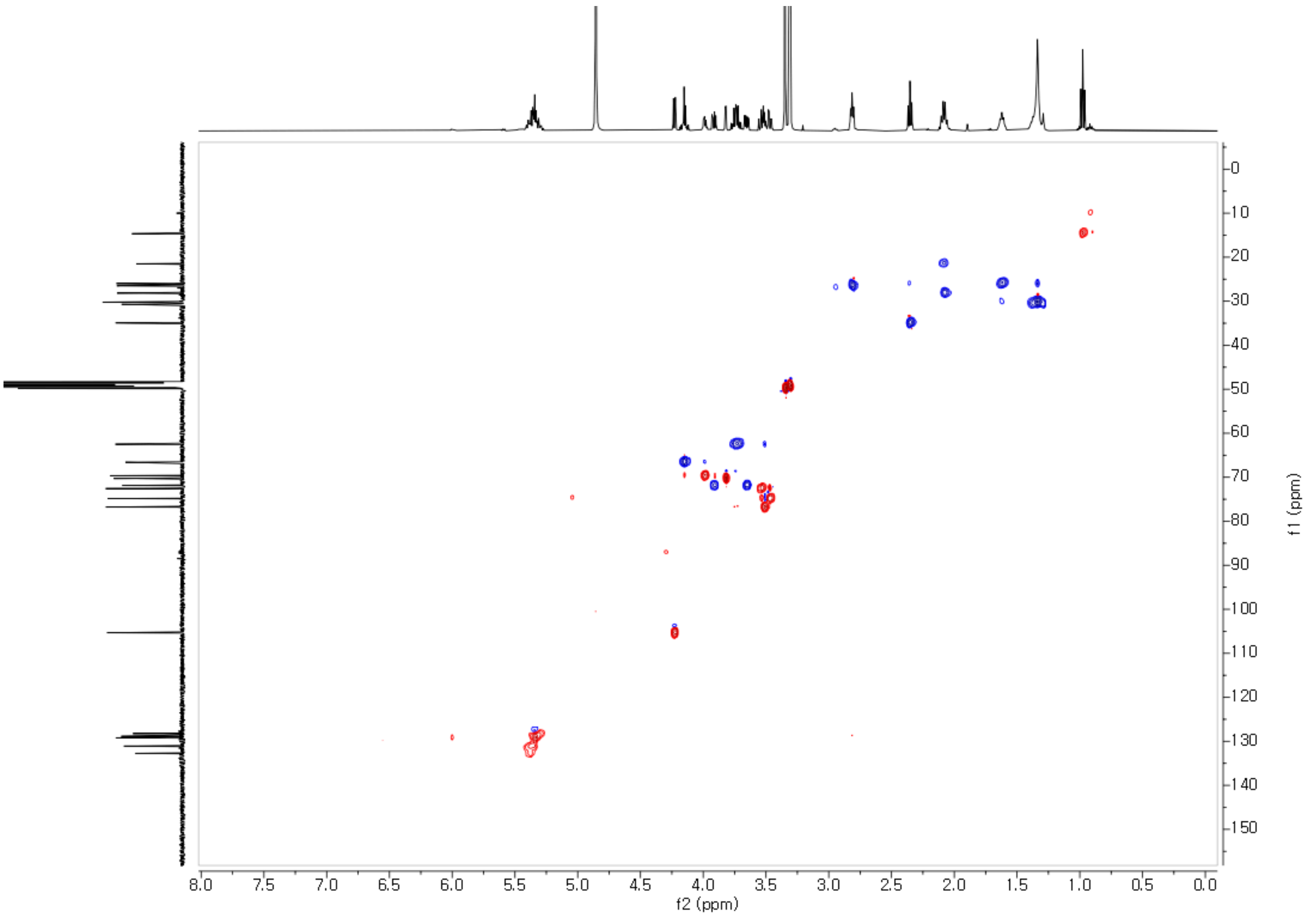

Figure S16. HSQC spectrum of compound **2-1** (methanol-d_4).

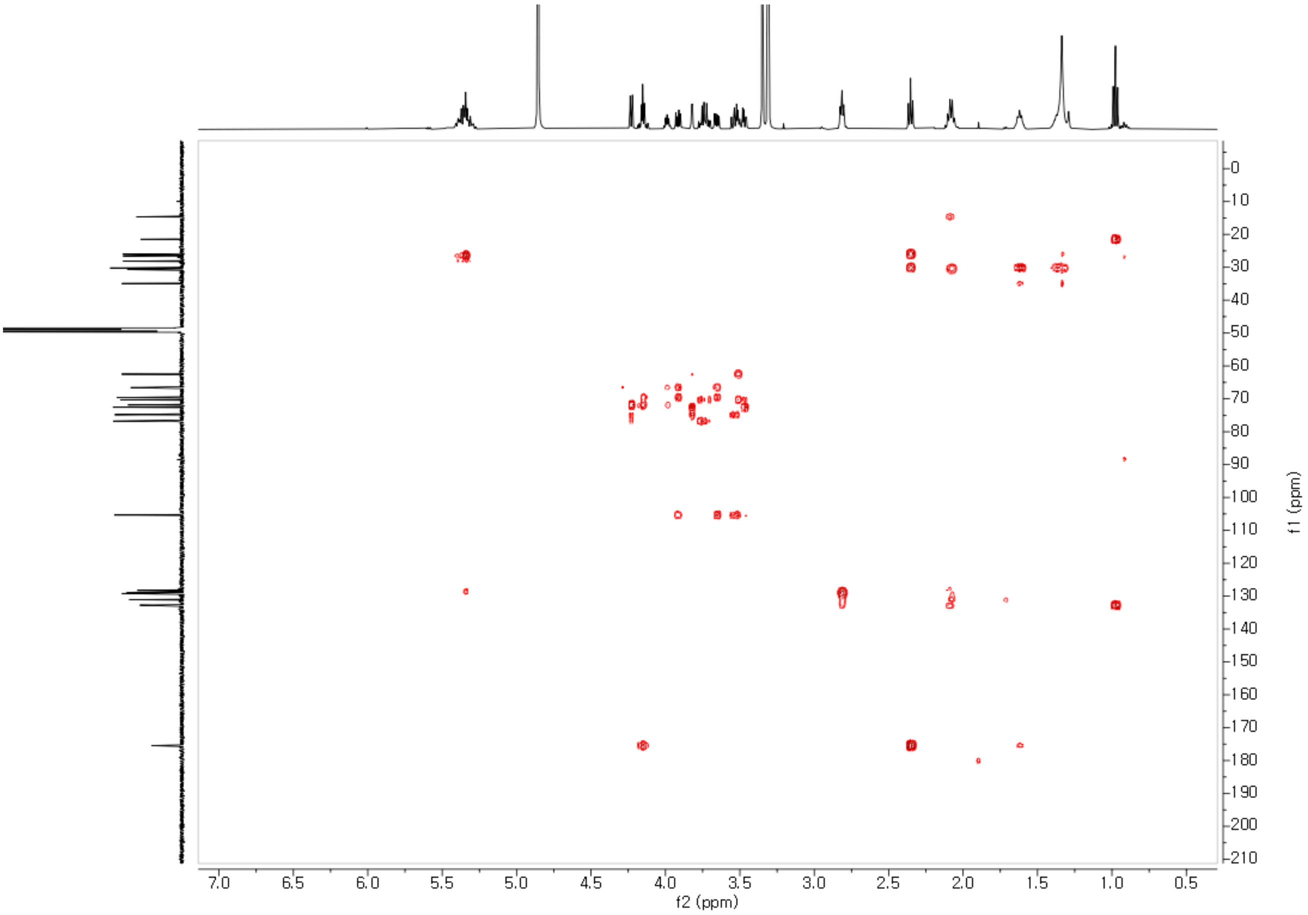

Figure S17. HMBC spectrum of compound **2-1** (methanol-*d*4).

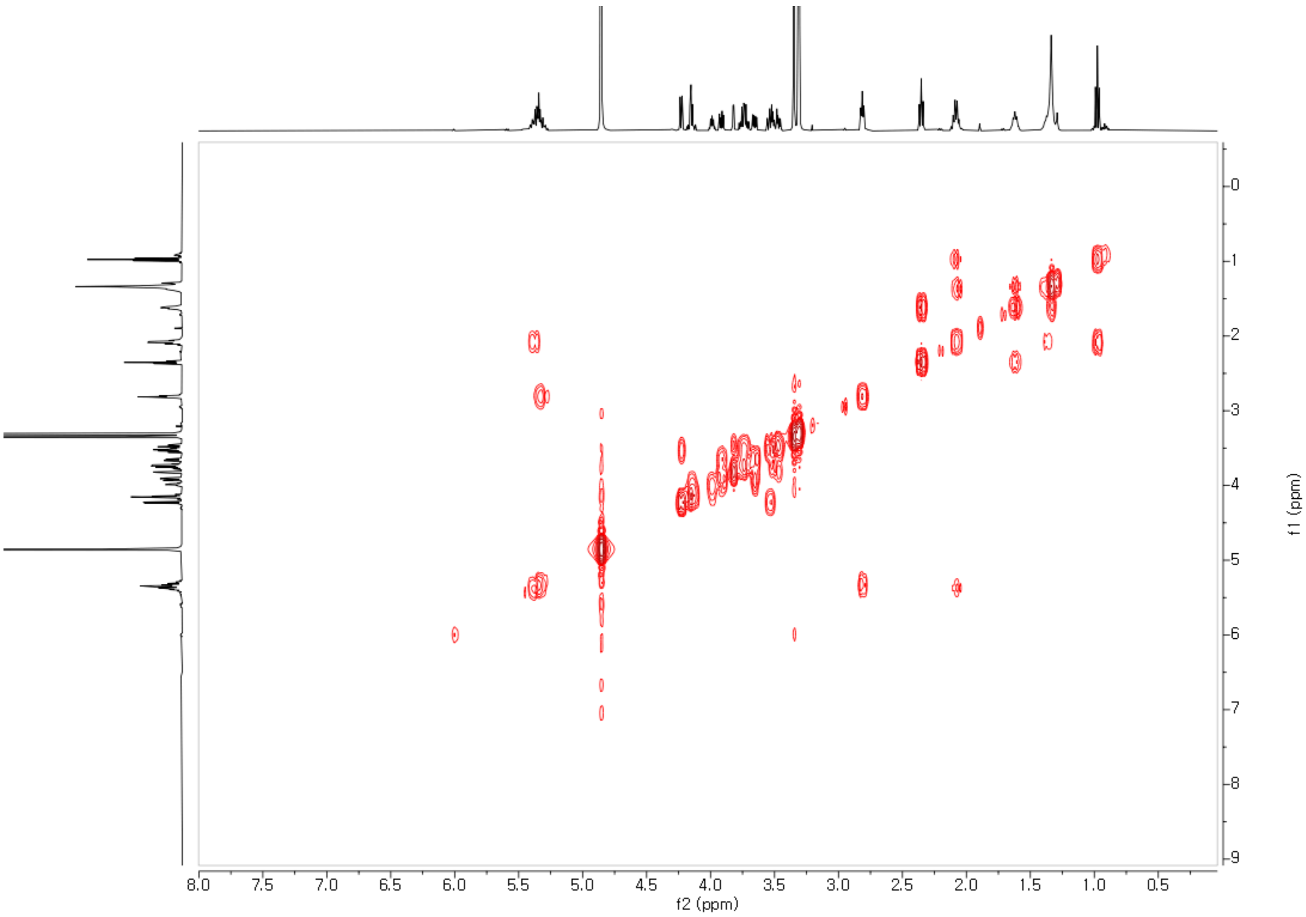

Figure S18. ^{1}H-^{1}H COSY spectrum of compound **2-1** (methanol-d_4).

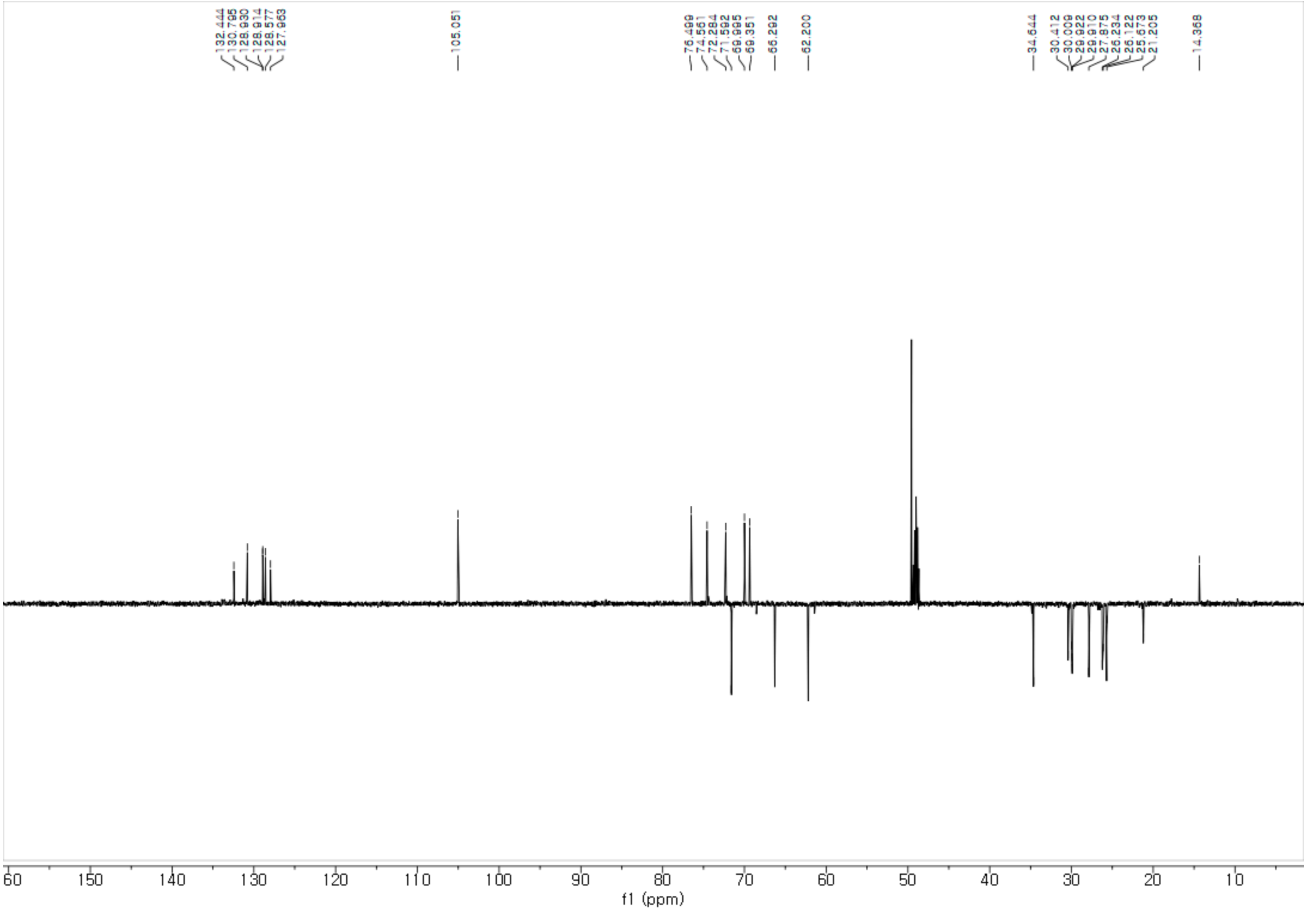

Figure S19. DEPT spectrum of compound **2-1** (methanol-d_4).

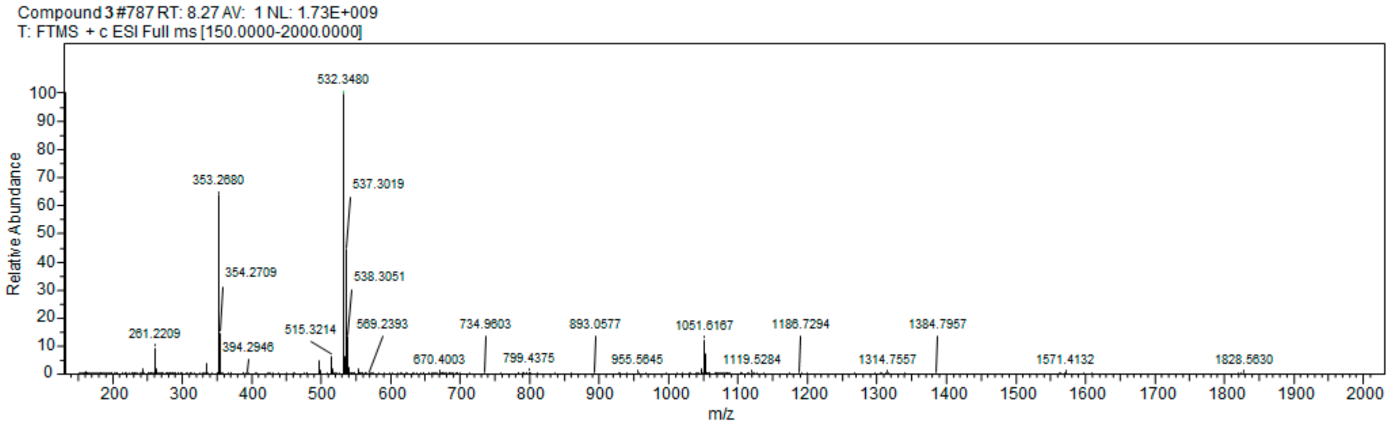

Peak Mass	Display Formula	Combined Fit	RDB	Delta [ppm]	Theo. mass	Rank	Combined Score	# Matched Iso.	# Missed Iso.	MS Cov. [%]	Pattern Cov. [%]
537.3019	$C_{27}H_{46}O_9{}^{23}Na$	50.31	4.50	-2.84	537.30340	1	97.38	5	0	100.00	100.00

Figure S20. HRESIMS spectrum of compound **2-1**.

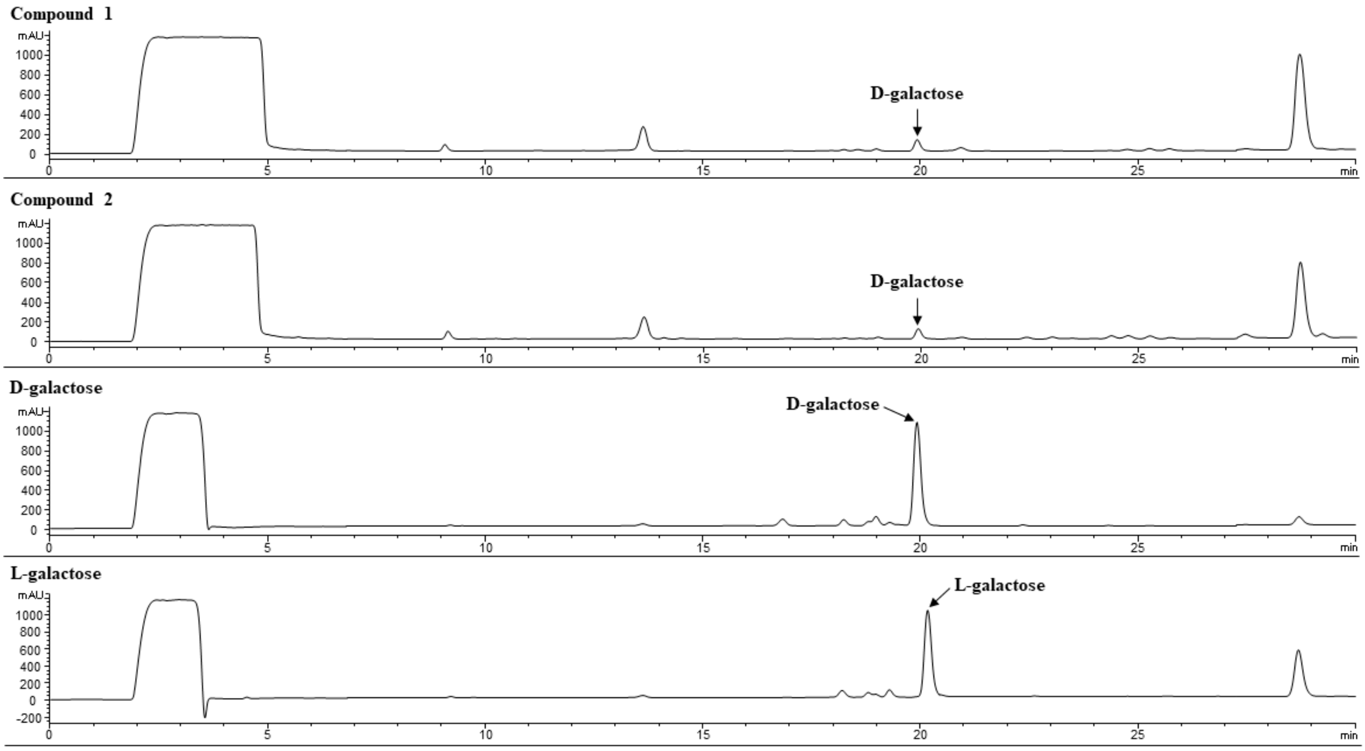

Figure S21. Sugar determination of compounds **1** and **2** by HPLC analysis.

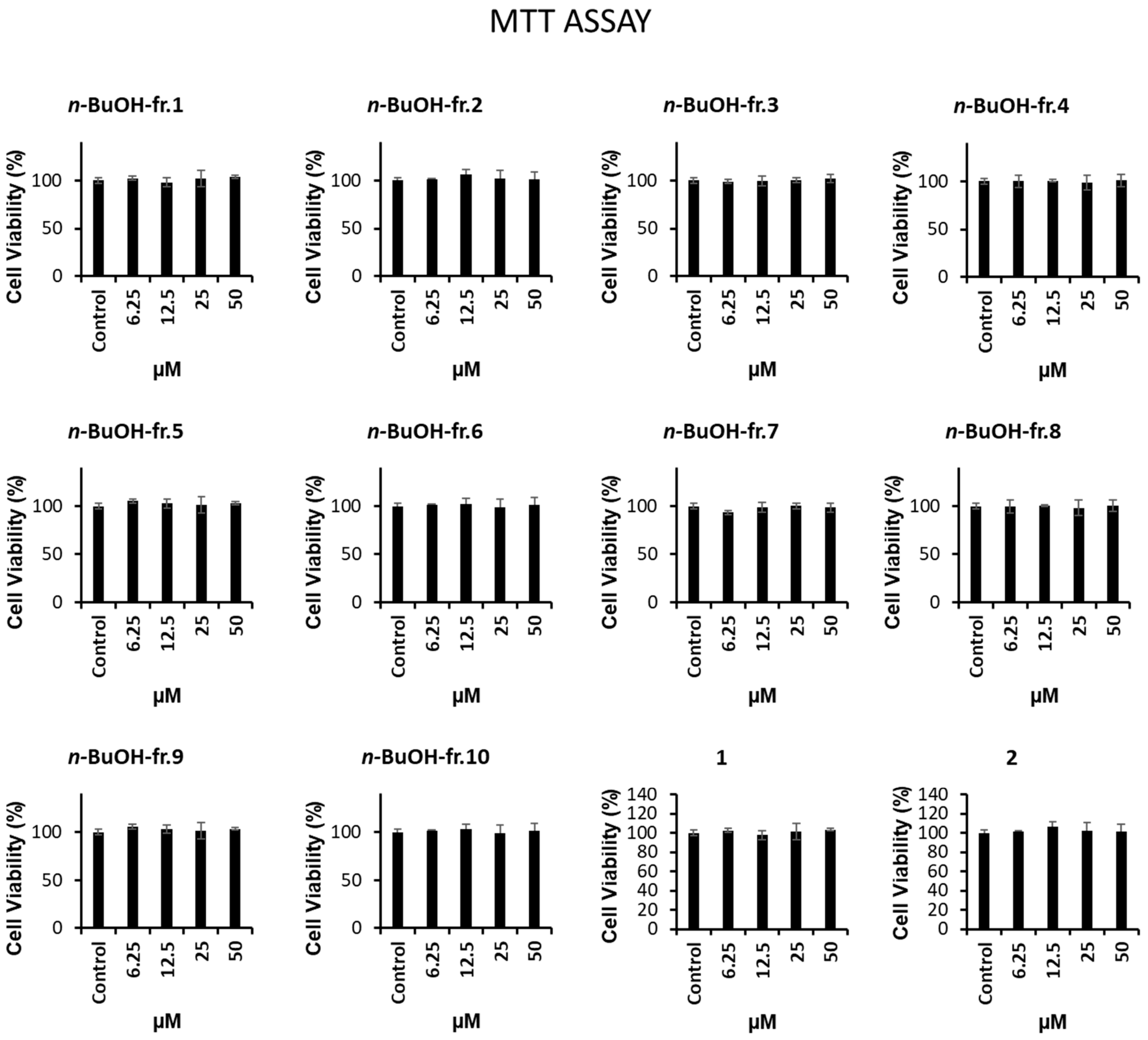

Figure S22. Cytotoxicity test of fractions and compounds
